# Usefulness of a Novel Mobile Diabetes Prevention Program Delivery Platform With Human Coaching: 65-Week Observational Follow-Up

**DOI:** 10.2196/mhealth.9161

**Published:** 2018-05-03

**Authors:** Andreas Michaelides, Jennifer Major, Edmund Pienkosz Jr, Meghan Wood, Youngin Kim, Tatiana Toro-Ramos

**Affiliations:** ^1^ Clinical Research Department Noom, Inc New York, NY United States; ^2^ Client Services Noom, Inc New York, NY United States; ^3^ Coaching Noom, Inc New York, NY United States; ^4^ Medicine Noom, Inc Seoul Republic Of Korea

**Keywords:** prediabetes, body weight, behavioral interventions, mHealth, mobile app, diabetes prevention

## Abstract

**Background:**

It is widely recognized that the prevalence of obesity and comorbidities including prediabetes and type 2 diabetes continue to increase worldwide. Results from a 24-week Diabetes Prevention Program (DPP) fully mobile pilot intervention were previously published showing promising evidence of the usefulness of DPP-based eHealth interventions on weight loss.

**Objective:**

This pilot study extends previous findings to evaluate weight loss results of core (up to week 16) and maintenance (postcore weeks) DPP interventions at 65 weeks from baseline.

**Methods:**

Originally, 140 participants were invited and 43 overweight or obese adult participants with a diagnosis of prediabetes signed up to receive a 24-week virtual DPP with human coaching through a mobile platform. At 65 weeks, this pilot study evaluates weight loss and engagement in maintenance participants by means of repeated measures analysis of variances and backward multiple linear regression to examine predictors of weight loss. Last observation carried forward was used for endpoint measurements.

**Results:**

At 65 weeks, mean weight loss was 6.15% in starters who read 1 or more lessons per week on 4 or more core weeks, 7.36% in completers who read 9 or more lessons per week on core weeks, and 8.98% in maintenance completers who did any action in postcore weeks (all *P*<.001). Participants were highly engaged, with 80% (47/59) of the sample completing 9 lessons or more and 69% (32/47) of those completing the maintenance phase. In-app actions related to self-monitoring significantly predicted weight loss.

**Conclusions:**

In comparison to eHealth programs, this pilot study shows that a fully mobile DPP can produce transformative weight loss. A fully mobile DPP intervention resulted in significant weight loss and high engagement during the maintenance phase, providing evidence for long-term potential as an alternative to in-person DPP by removing many of the barriers associated with in-person and other forms of virtual DPP.

## Introduction

It is widely recognized that the prevalence of obesity and comorbidities such as prediabetes and type 2 diabetes continue to increase worldwide [1,2]. In the United States, 84.1 million persons have prediabetes while 30.3 million have diabetes [3]. Recent reports show 35% of men and 40% of women with obesity [4]. Large longitudinal clinical trials have shown the effectiveness of lifestyle interventions and behavior change for weight loss and type 2 diabetes risk reduction [5-10], performing better than medication. The original National Diabetes Prevention Program (NDPP) intervention study provided an intensive lifestyle intervention focused on weight loss of at least 7% of body weight through a low-fat diet and a minimum increase of 150 minutes of weekly physical activity [7]. The intervention resulted in reduced incidence of diabetes of 58% at 3 years [7]. At 15 years’ follow-up, incidence was reduced by 27% in the intensive lifestyle intervention group [5]. Therefore, lifestyle and behavior interventions have become the standard recommendation for risk reduction in persons with overweight or obesity [11]. Lifestyle in-person programs can be very effective, but the cost may be unsustainable [12]. With the advent of digital technology, evidence points to the noninferiority and at times superiority of mHealth and eHealth, telecommunications translations of lifestyle interventions such as the Diabetes Prevention Program (DPP) [13] to prevent and manage chronic conditions.

A recent review of DPP-based lifestyle interventions via electronic, mobile, and telehealth or eHealth interventions with remote counseling up to 15 months from baseline, looking at predominantly white and educated samples, found mean sustained weight loss of 4.31% compared to 4.65% with a counselor in-person [13]. There is promising evidence of the usefulness of DPP-based eHealth interventions on weight loss. In participants over 65 years, a single-arm study showed 7.5% weight loss at 12 months for those who completed a 12-month program with a Centers for Disease Control and Prevention (CDC)-based DPP with human coaching [14].

We previously reported findings from a 6-month fully mobile DPP intervention in a group of overweight and obese hyperglycemic adults [15]. Briefly, at 24 weeks core completers (84% of the sample) lost 7.5% of body weight. The purpose of this pilot study is to report 65-week weight loss from baseline and at completion of the maintenance component of the mobile DPP intervention. We hypothesized that clinically and statistically significant weight loss at 65 weeks would be maintained (>5% body weight).

## Methods

### Recruitment

A detailed intervention description was previously published [15]. Briefly, participants were recruited by email or regular mail by a large Northeast-based insurance company that offered its employees the Noom app free of cost; employees did not receive additional compensation for their participation. The email or regular mail invitation contained information describing the study. Potential participants across different departments and states were included if they had an elevated hemoglobin A_1c_ (HbA_1c_) status (5.7% to 6.4%) in their medical records, reflecting a diagnosis of prediabetes. Interested participants were assigned to a virtual NDPP coach who had successfully completed a CDC-recognized training course.

**Figure 1:**
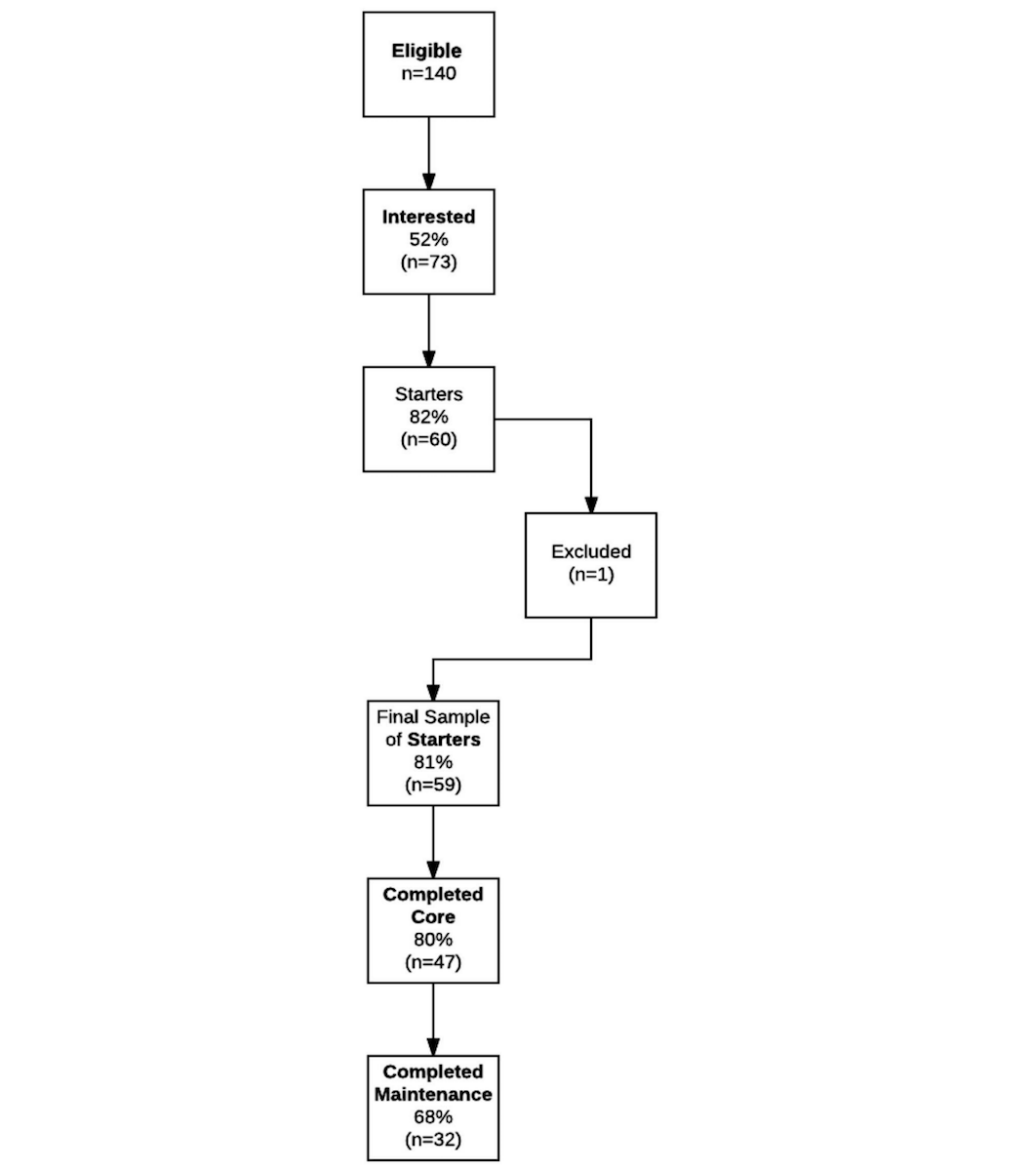
Flowchart for study recruitment, starting, and completion or core and maintenance status.

Men and women with an HbA_1c_ of 5.7% to 6.4% between the ages of 18 and 75 years who signed up for Noom’s NDPP program (Figure 1, n=140) between June 22 and September 7 (recruitment phase 1 as previously reported [15]) and September 8 and November 30, 2015 (phase 2 added 19 participants), were included in the study. Out of 140, individuals were considered interested (n=73) if they performed at least 1 in-app action during the first week of the NDPP curriculum. Sixty-seven participants did not perform any in-app actions during the first week of the NDPP curriculum and were not included in the study. From 73 interested participants, those having <2 weigh-ins (n=13) and who did not read more than one article per week for 4 or more weeks were considered nonstarters and were excluded from final analyses. Participants who attended (ie, read content) during at least any 4 of the initial 16 weeks were considered to have started, in accordance with the CDC’s definition (n=60) [16]. We excluded a single participant because that person failed to provide more than 1 weigh-in, thus resulting in 59 participants making up our final sample of starters. Out of the starters, participants were considered completers (n=47) if they attended any 9 of the 16 weeks of the core program [16]. Only participants who completed the core phase were eligible for the maintenance phase, and those who attended at least 7 maintenance sessions were considered maintenance phase completers (n=32). As multiple maintenance session topics are delivered each month, attendance at 7 sessions exceeded the 50% minimum Diabetes Prevention Recognition Program standard [16].

**Figure 2:**
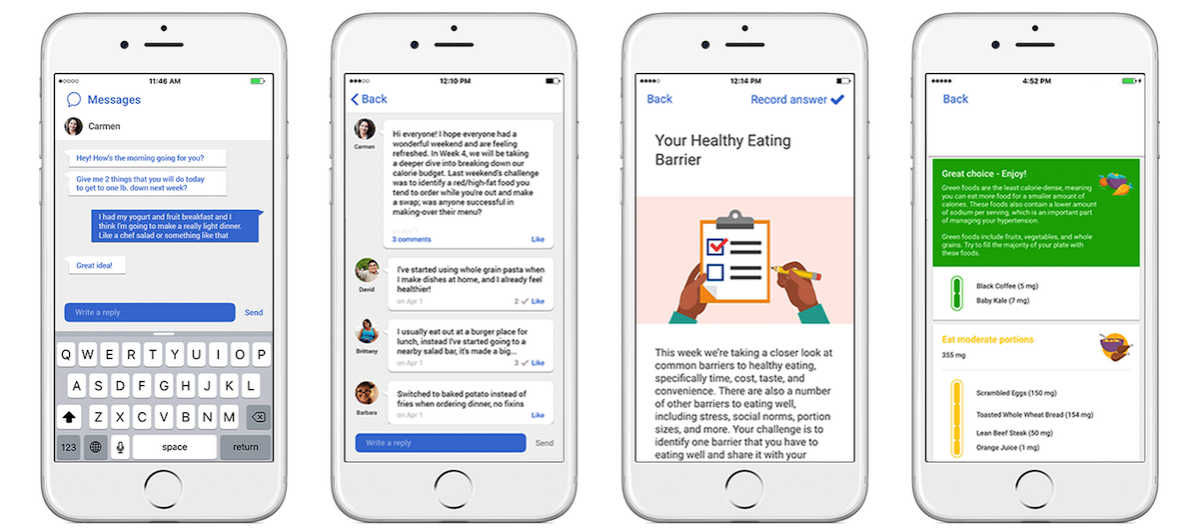
The Noom Coach app's interactive interface with coach-user messaging, group messaging, daily challenges, food logging with color coding and automated feedback based on food choices.

During the first week of the study, participants received orientation on what the DPP program entails, learned how to use the Noom app, how to interact with their coach, and the importance of maintaining motivation throughout the program. The Noom Coach app (Figure 2) offers an interactive interface with coach-participant messaging, group messaging, daily challenges for behavior change, the DPP-based education articles, food logging with color coding, and automated feedback based on food choices. Participants received daily DPP content through informative articles and were asked to log their weight by self-report, meals, and physical activity within the app on a weekly basis. NDPP-certified coaches securely monitored participant progress through a dashboard. The emphasis of the intervention is on lifestyle change and finding a system of healthy living that fits into the context of each user’s individual life. Therefore, the objective was that the participant interacted with the coach, the group, and the app in general in a way that seamlessly integrated into their life. Participants were instructed to communicate as much or as little as needed to support their individual journey. Participants were informed that they could expect to hear from their coach every day (an individual message or a message addressed to the entire group). Basic instructions encompassed the message “You get out of it what you put into it. Be as present as you can in the app. Log as much as possible, as this gives me (your coach) insight into your health habits and gives context to all discussions. The more engaged you are with the app, the more likely you will be to stay on track toward your health goals.”

Participants engaged in the program by completing actions that included meals logged (meals per week), green foods logged (logged per week), exercise logged (times per week), exercise time registered (minutes per week), steps recorded (steps per week), weigh-ins logged (times per week), articles read (articles per week), group posts (posts per week), group comments (comments per week), messages sent to their coach (messages per week), and group likes (likes per week). This pilot study reports 65-week results including the maintenance DPP phase.

### Statistical Analysis

Descriptive statistics, expressed as means and standard deviations or frequencies and percentages for continuous and categorical variables, respectively, were calculated for subject baseline characteristics. Mean weekly in-app action variables were calculated as means and standard deviations from baseline to 65 weeks for starters, completers, and maintenance completers. Last observation carried forward was utilized for endpoints. Repeated measures analysis of variance examined the effect size of the change in weight and body mass index (BMI) from baseline to 24 weeks and 65 weeks in starters, completers, and maintenance completers based on the CDC standards [16]. Multiple linear backward regression analyses examined whether in-app engagement variables predicted weight loss at 65 weeks. This method was used to evaluate which variables accounted for the most variance in the model, removing those that did not render a good fit and providing insight into what user behaviors predicted weight loss. Significance tests are 2-sided with a significance of *P*<.05. Statistical analyses were performed using SPSS Statistics version 21 (IBM Corp) software.

## Results

At baseline, 81% (48/59) of the sample was female with a mean BMI of 34 kg/m^2^ and mean age of 51 years (Table 1). Relative to baseline, starters lost 5.93 kg at 65 weeks and BMI was reduced by 2.12 kg/m^2^. Among completers, weight loss was 7.05 kg and BMI change was –2.53 kg/m^2^. Maintenance completers lost 8.78 kg of body weight and BMI was reduced by 3.14 kg/m^2^, all *P*<.001. Among starters, 44% (26/59) lost ≥5% body weight while 53% (25/47) of completers and 66% (21/32) of maintenance completers lost ≥5% body weight, respectively (Table 2).

In maintenance completers, mean body weight decreased significantly by 7.64 kg between baseline and 24 weeks and was sustained over time by 8.77 kg from baseline to 65 weeks (both *P*<.001). Weight change at 65 weeks was not different to that at 24 weeks (–1.13 kg, *P*=.29) (Table 3). BMI at 24 and 65 weeks did not differ and was significantly lower than at baseline (Table 3). Raw data are shown in Multimedia Appendix 1.

Mean in-app engagement variables are shown in Table 4. Multiple linear backward regression examined in-app engagement variables as predictors of weight loss controlling for age and sex (Multimedia Appendix 2). The variables that remained in the model were mean weekly logged meals, exercise, articles read, group posts, and messages to coach. Participants with higher engagement and self-monitoring through meal logging and group posts had higher weight loss, whereas messaging the coach, logging exercise, and reading articles predicted weight gain.

**Table 1:** Baseline characteristics of study starters, completers, and maintenance completers. BMI: body mass index.

Characteristics	Starters (n=59)	Completers (n=47)	Maintenance completers (n=32)
Gender, female, n (%)	48 (81)	37 (79)	26 (81)
Age, years, mean (SD)	51.27 (9.25)	51.45 (9.47)	51.34 (9.55)
Height, cm, mean (SD)	166.75 (9.38)	167.62 (9.16)	167.44 (9.04)
Weight, kg, mean (SD)	95.36 (22.11)	95.66 (23.10)	96.83 (24.94)
BMI, kg/m^2^, mean (SD)	34.19 (6.75)	33.90 (6.83)	34.39 (7.41)

**Table 2:** Weight loss from baseline to 65 weeks, *P*<.001. BMI: body mass index.

Characteristics	Starters (n=59)	Completers (n=47)	Maintenance completers (n=32)
Weight change, kg, mean (SD)	–5.93 (6.78)	–7.05 (7.10)	–8.78 (7.71)
Weight change, %, mean (SD)	–6.15 (6.50)	–7.36 (6.67)	–8.98 (7.12)
BMI change, kg/m^2^, mean (SD)	–2.12 (2.43)	–2.53 (2.54)	–3.14 (2.76)

**Table 3:** Weight change from baseline to 24 weeks and 65 weeks in maintenance completers (n=32). BMI: body mass index.

Characteristics	Baseline	24 weeks	*P* value^a^	65 weeks	*P* value^b^
Weight, kg, mean (SD)	96.83 (4.41)	89.18 (4.29)	<.001	88.06 (4.27)	.29
BMI, kg/m^2^, mean (SD)	34.39 (7.41)	31.66 (7.22)	<.001	31.24 (7.11)	.28

^a^Change from baseline to 24 and 65 weeks.

^b^Change from 24 weeks to 65 weeks.

**Table 4:** Mean in-app^a^ weekly engagement variables over 65 weeks.

Weekly engagement variable	Starters (n=59)		Completers (n=47)		Maintenance completers (n=32)	
	n^b^	mean (SD)	n	mean (SD)	n	mean (SD)
Meals logged (meals/week)^c^	57	15.28 (3.99)	45	15.89 (3.87)	30	16.39 (3.26)
Green foods (logged/week)^d^	59	0.27 (.13)	47	0.27 (0.13)	32	0.29 (0.14)
Exercise (times/week)	57	4.52 (2.70)	47	4.60 (2.34)	32	4.70 (2.07)
Time exercised (minutes/week)	57	169.68 (113.93)	47	175.06 (111.06)	32	188.65 (119.18)
Steps recorded (steps/week)	52	23,427.95 (14,709.14)	43	24,397.80 (15,311.71)	29	23,696.13 (13,845.60)
Number of weigh-ins (times/week)^e^	59	1.40 (0.85)	47	1.48 (0.93)	32	1.53 (0.99)
Articles read (articles/week)	59	8.12 (3.34)	47	8.59 (2.97)	32	8.56 (2.73)
Group posts (posts/week)^f^	53	1.88 (1.10)	43	1.91 (1.13)	30	1.93 (1.14)
Group comments (comments/week)^g^	53	2.85 (1.52)	44	2.90 (1.46)	30	3.08 (1.40)
Messages to coach (messages/week)	56	3.92 (1.67)	45	4.09 (1.96)	31	4.48 (1.83)
Group likes (likes/week)^h^	49	2.34 (1.46)	42	2.28 (1.55)	30	2.33 (1.76)

^a^In Michaelides et al [15], it was previously reported that in-app actions related to self-monitoring over 24 weeks significantly predicted weight loss.

^b^n represents the number of starters, completers, or maintenance completers that engaged in with the app feature.

^c^Meals logged refers to the times breakfast, lunch, snack, and dinner were logged per week.

^d^Green foods refers to food items logged that contain low calorie density (calories per grams in a serving).

^e^Number of weigh-ins refers to times per week of in-app weight self-reports.

^f^Group posts refers to times per week a participant posted to their group in an in-app common conversation.

^g^Group comments refers to responses to group posts per week.

^h^Group likes refers to times per week a participant liked a group comment.

## Discussion

### Principal Findings

This 65-week pilot study shows that a mobile DPP intervention is capable of producing weight loss outcomes that are comparable to those seen in in-person NDPP or similar virtual programs [7,17,18]. Core completers represented 80% of the final sample (47/59), and maintenance completers represented 69% of core completers (32/47). Core completers lost 7.36% of body weight and maintenance completers lost 8.98%, comparable to outcomes seen in other observational DPP studies. Findings suggest that a mobile DPP program can facilitate transformative weight loss in participants as well as significant weight maintenance after 1 year. At 6 months, in-app engagement predicted weight loss, with key indicators including weekly weigh-ins, meals logged, and weekly posts to the group [15]. In the same study, those who made more group posts also logged more meals, and meal logging partially mediated the relationship between group posts and percentage weight loss [15]. Follow-up results at 65 weeks in this maintenance study did not show mediation but further support the conclusions from the 6-month findings [15]. Mean weekly logged meals, exercise, articles read, group posts, and messages to coach predicted weight loss. Participants with higher engagement and self-monitoring through meal logging and group posts had higher weight loss, whereas messaging the coach, logging exercise, and reading articles predicted weight gain. It is known that monitoring weight and following structured lessons are predictors of weight loss [19] and sustained weight loss over time. In this pilot study, some engagement variables were correlated, potentially leading to collinearity and contradiction among variable predictions (reading articles, messaging the coach, and exercising more) in the model. Another interpretation, however, could be that users who reach out to their coach more frequently may be struggling more with weight loss compared with those who reach out less and manage weight loss more effectively. This warrants further investigation in a larger sample and shows that a platform that combines a low-barrier self-monitoring method is highly relevant to successful facilitation of a virtual DPP.

### Comparison With Prior Work

While there has been extensive research on eHealth approaches to diabetes prevention [13,20-24], to our knowledge this study is the first fully mobile (mHealth) translation of the DPP with more than 1-year follow-up. This study shows that a fully mobile DPP is similar in producing desired weight loss outcomes in comparison with Web-based or telehealth programs. These findings are particularly significant given that the use of mobile phones is expected to reach over one-third of the world’s population by 2018 [25].

At 65 weeks, maintenance completers lost 8.98% of their starting weight, which exceeds the CDC’s NDPP requirement of 5% loss of body weight at 6 months and 12 months [3]. Other eHealth interventions for diabetes prevention have reached different levels of weight loss at 12 months. For example, Fisher et al [26] found a 2.31 kg weight loss in the intervention group of a text message–based intervention, and Sepah et al [18] found a 5.13 kg weight loss at 12 months in a Web-based intervention [18], while our study showed a mean weight loss of 7.05 and 8.78 kg in core completers and maintenance phase completers, respectively, at 65 weeks.

Furthermore, in comparison with recent in-person DPP trials, which have scalability and cost-effectiveness challenges [27], results indicate that weight loss outcomes may even be higher in virtual DPP [28,29]. One possible reason for these outcomes is that virtual DPP allows for a participant to interact with a group and facilitator more than once per week. Daily delivery of content combined with real-time access to facilitators has resulted in greater engagement and longer retention when compared to in-person or digital programs offered once per week. This is supported by the positive association between engagement metrics and weight loss outcomes seen in our results. Furthermore, the assumption that virtual DPP programs can be just as, or more, effective than in-person DPP programs is supported by virtual DPP research beyond this pilot. A similar single-arm trial resulted in participants losing 7.5% of body weight [14] while meta-analyses have also shown promising evidence of the efficacy of virtual DPP programs [13,20].

### Limitations

While the results are promising, there are limitations to this pilot study. As this is a pilot study, the sample size is small (n=59). Furthermore, this was an observational study; thus, the participants were self-selected and likely highly motivated individuals, resulting in limited generalizability. Nonetheless, per observational study design, participants were both self-selected and self-monitored, which mimics real-world applications of a virtual DPP program. An additional limitation is the lack of HbA_1c_ values. However, the main outcome of the DPP is weight loss above the transformative value of 5% as established by the CDC. Medication history, which could influence weight loss, was not collected. Participant/coach and group interactions were assessed quantitatively, which could have provided more insight into participant attitudes and behaviors and better explain weight loss and behavior change, instead of qualitatively. To validate the findings of this pilot study, future long-term randomized controlled trials should aim to include biometric data such as HbA_1c_ level and quality of life metrics.

### Conclusions

This pilot study of a fully mobile DPP intervention found significant weight loss and high engagement during the maintenance phase, providing evidence for long-term potential as an alternative to in-person DPP by removing many of the barriers associated with in-person and other forms of virtual DPP.

## Appendix

Multimedia Appendix 1 Raw weight change data at baseline, 24 weeks, and 65 weeks for maintenance completers.

Multimedia Appendix 2 Backward multiple linear regression of engagement variables as predictors of weight loss in maintenance completers.

## Abbreviations

BMI: body mass index
CDC: Centers for Disease Control and Prevention
DPP: diabetes prevention program
HbA _1c_: hemoglobin A_1c_
NPDD: National Diabetes Prevention Program
