# Supplementary figures and images for: Usefulness of a Novel Mobile Diabetes Prevention Program Delivery Platform With Human Coaching: 65-Week Observational Follow-Up
Source: JMIR Mhealth Uhealth. 2018 May 3;6(5):e93. doi: 10.2196/mhealth.9161 (PMC5958286; doi:10.2196/mhealth.9161)

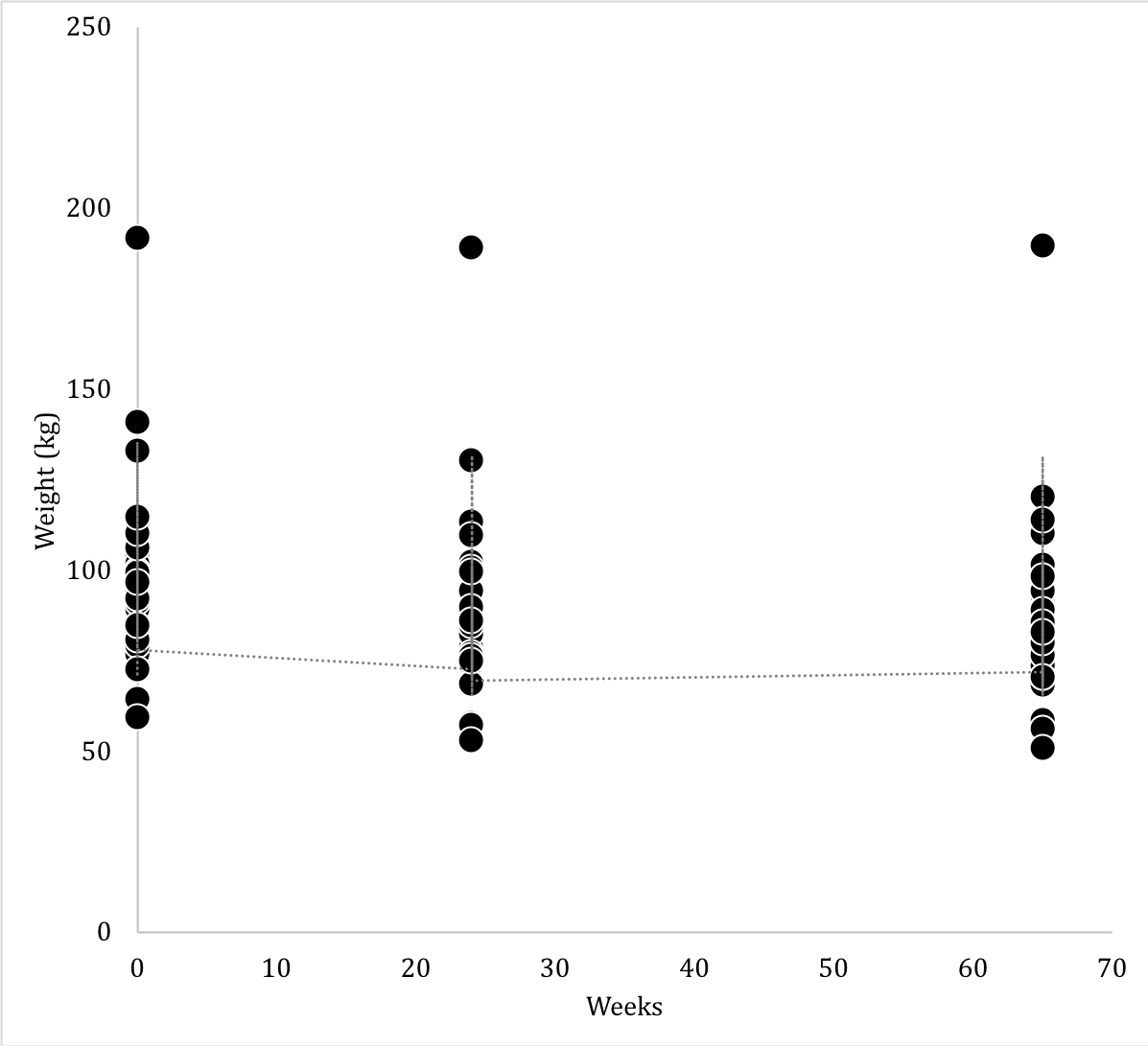

Supplement: Multimedia Appendix 1 [file mhealth_v6i5e93_app1.png]
