# Supplementary material for: Usefulness of a Novel Mobile Diabetes Prevention Program Delivery Platform With Human Coaching: 65-Week Observational Follow-Up
Source: JMIR Mhealth Uhealth. 2018 May 3;6(5):e93. doi: 10.2196/mhealth.9161 (PMC5958286; doi:10.2196/mhealth.9161)
Supplement: Multimedia Appendix 2 [file mhealth_v6i5e93_app2.pdf]

Multimedia Appendix 2. Backward multiple linear regression of engagement variables as predictors of weight loss in maintenance completers.

Model		Beta	t	P	Tolerance	VIF	R	R Square	Adj. R Square	F	P
1	(Constant)		-0.22	.827			0.83	0.70	0.30	1.76	.188
	Gender	0.13	0.53	.606	0.48	2.09					
	Age (years)	0.01	0.05	.962	0.45	2.25					
	Meals logged (meals per week)[b]	-0.83	-2.31	.043	0.24	4.22					
	Green Foods (logged per week)[c]	-0.02	-0.09	.933	0.49	2.04					
	Exercise (times/week)	0.30	0.77	.458	0.20	5.01					
	Time exercised (minutes/week)	0.12	0.37	.721	0.28	3.52					
	Steps Recorded (steps/week)	-0.19	-0.82	.429	0.57	1.76					
	Number of weigh-ins (times/week)[d]	-0.19	-0.56	.59	0.25	3.99					
	Articles read (articles/week)	0.64	2.40	.037	0.43	2.34					
	Group posts (posts/week)[e]	-0.95	-2.76	.02	0.26	3.90					
	Group comments (comments/week)[f]	0.13	0.29	.774	0.17	6.05					
	Group likes (likes/week)[g]	0.13	0.34	.	0.22	4.5					

				743		7					
	Messages to coach (messages/week)	0.86	2.65	.025	0.29	3.47					
2	(Constant)		-0.25	.806			0.83	0.70	0.36	2.10	.115
	Gender	0.14	0.61	.556	0.54	1.87					
	Meals logged (meals per week)[b]	-0.82	-2.63	.024	0.28	3.54					
	Green Foods (logged per week)[c]	-0.02	-0.09	.929	0.49	2.04					
	Exercise (times/week)	0.30	0.81	.436	0.20	4.99					
	Time exercised (minutes/week)	0.12	0.38	.71	0.29	3.49					
	Steps Recorded (steps/week)	-0.19	-0.87	.402	0.59	1.69					
	Number of weigh-ins (times/week)[d]	-0.20	-0.66	.521	0.30	3.32					
	Articles read (articles/week)	0.64	2.54	.027	0.44	2.28					
	Group posts (posts/week)[e]	-0.95	-2.93	.014	0.26	3.84					
	Group comments (comments/week)[f]	0.12	0.31	.765	0.18	5.66					
	Group likes (likes/week)[g]	0.13	0.36	.726	0.22	4.54					
	Messages to coach (messages/week)	0.86	2.86	.016	0.30	3.29					
3	(Constant)		-0.29	.774			0.83	0.70	0.42	2.49	.066
	Gender	0.14	0.63	.541	0.54	1.86					

	Meals logged (meals per week)[b]	-0.82	-2.74	.018	0.28	3.52					
	Exercise (times/week)	0.29	0.87	.402	0.23	4.35					
	Time exercised (minutes/week)	0.13	0.44	.667	0.31	3.24					
	Steps Recorded (steps/week)	-0.19	-0.91	.379	0.59	1.69					
	Number of weigh-ins (times/week)[d]	-0.20	-0.70	.497	0.30	3.30					
	Articles read (articles/week)	0.64	2.66	.021	0.44	2.28					
	Group posts (posts/week)[e]	-0.95	-3.10	.009	0.27	3.68					
	Group comments (comments/week)[f]	0.10	0.33	.746	0.28	3.53					
	Group likes (likes/week)[g]	0.14	0.44	.667	0.25	3.93					
	Messages to coach (messages/week)	0.87	3.01	.011	0.31	3.26					
4	(Constant)		-0.26	.8			0.83	0.69	0.46	2.93	.036
	Gender	0.13	0.61	.551	0.55	1.83					
	Meals logged (meals per week)[b]	-0.81	-2.82	.014	0.29	3.45					
	Exercise (times/week)	0.28	0.88	.396	0.23	4.33					
	Time exercised (minutes/week)	0.12	0.44	.666	0.31	3.23					
	Steps Recorded (steps/week)	-0.20	-1.00	.337	0.60	1.67					
	Number of weigh-ins	-0.23	-0.86	.	0.33	3.0					

	(times/week)[d]			404		2					
	Articles read (articles/week)	0.65	2.81	.015	0.44	2.25					
	Group posts (posts/week)[e]	-0.92	-3.28	.006	0.30	3.31					
	Group likes (likes/week)[g]	0.20	0.85	.41	0.41	2.43					
	Messages to coach (messages/week)	0.87	3.15	.008	0.31	3.25					
5	(Constant)		-0.31	.761			0.83	0.69	0.49	3.43	0.019
	Gender	0.11	0.56	.588	0.57	1.76					
	Meals logged (meals per week)[b]	-0.79	-2.87	.012	0.29	3.40					
	Exercise (times/week)	0.38	1.67	.118	0.44	2.30					
	Steps Recorded (steps/week)	-0.19	-0.98	.342	0.61	1.65					
	Number of weigh-ins (times/week)[d]	-0.31	-1.65	.12	0.63	1.59					
	Articles read (articles/week)	0.64	2.86	.013	0.45	2.23					
	Group posts (posts/week)[e]	-0.94	-3.51	.003	0.31	3.20					
	Group likes (likes/week)[g]	0.23	1.01	.332	0.43	2.31					
	Messages to coach (messages/week)	0.91	3.55	.003	0.34	2.94					
6	(Constant)		0.01	.996			0.83	0.68	0.51	4.01	0.01
	Meals logged (meals per week)[b]	-0.84	-3.24	.005	0.32	3.12					

	Exercise (times/week)	0.43	2.11	.053	0.52	1.94					
	Steps Recorded (steps/week)	-0.21	-1.13	.275	0.63	1.59					
	Number of weigh-ins (times/week)[d]	-0.34	-1.87	.082	0.66	1.52					
	Articles read (articles/week)	0.65	3.01	.009	0.45	2.21					
	Group posts (posts/week)[e]	-0.94	-3.60	.003	0.31	3.20					
	Group likes (likes/week)[g]	0.24	1.07	.303	0.44	2.30					
	Messages to coach (messages/week)	0.88	3.61	.003	0.36	2.77					
7	(Constant)		0.52	.609			0.81	0.66	0.51	4.38	.007
	Meals logged (meals per week)[b]	-0.88	-3.44	.003	0.33	3.04					
	Exercise (times/week)	0.43	2.11	.051	0.52	1.94					
	Steps Recorded (steps/week)	-0.19	-1.04	.314	0.63	1.58					
	Number of weigh-ins (times/week)[d]	-0.26	-1.57	.137	0.78	1.28					
	Articles read (articles/week)	0.64	2.95	.009	0.45	2.21					
	Group posts (posts/week)[e]	-0.77	-3.76	.002	0.52	1.94					
	Messages to coach (messages/week)	0.73	3.62	.002	0.53	1.90					
8	(Constant)		0.58	.571			0.80	0.63	0.51	4.91	.004
	Meals logged (meals per week)[b]	-0.92	-3.65	.	0.34	2.9					

				002		6					
	Exercise (times/week)	0.43	2.12	.049	0.52	1.94					
	Number of weigh-ins (times/week)[d]	-0.22	-1.37	.19	0.82	1.22					
	Articles read (articles/week)	0.63	2.88	.011	0.46	2.19					
	Group posts (posts/week)[e]	-0.70	-3.60	.002	0.56	1.77					
	Messages to coach (messages/week)	0.61	3.65	.002	0.76	1.31					
9	(Constant)		0.62	.546			0.77	0.59	0.48	5.26	.004
	Meals logged (meals per week)[b]	-0.96	-3.73	.002	0.34	2.93					
	Exercise (times/week)	0.36	1.79	.09	0.55	1.82					
	Articles read (articles/week)	0.65	2.91	.009	0.46	2.18					
	Group posts (posts/week)[e]	-0.66	-3.34	.004	0.58	1.72					
	Messages to coach (messages/week)	0.55	3.33	.004	0.82	1.22					

[a]In Michaelides et al 2016, it was previously reported that in-app actions related to self-monitoring over 24 weeks significantly predicted weight loss [15].

[b]Meals logged refer to the times breakfast, lunch, snack and dinner were logged per week.

[c]Green foods refer to food items logged that contain low calorie density (calories per grams in a serving).

[d]Number of weigh-ins are times per week of in-app weight self-reports.

[e]Group posts are times per week a participant posted to their group in an in-app common conversation while [f]group comments are responses to group posts per week, and [g]group likes refer to the times per week a participant liked a group comment.
